# Ecological Niche Differentiation Parallels Genomic Divergence of Three *Thelephora* Ectomycorrhizal Fungi

**DOI:** 10.3390/jof12090673

**Published:** 2026-09-08

**Authors:** Si-Ao Li, Hyang Burm Lee, Hai-Sheng Yuan

**Affiliations:** 1 CAS Key Laboratory of Forest Ecology and Silviculture, Institute of Applied Ecology, Chinese Academy of Sciences, Shenyang 110164, China; li_siao@163.com; 2 University of Chinese Academy of Sciences, Beijing 100049, China; 3 Environmental Microbiology Lab, Department of Agricultural Biological Chemistry, College of Agriculture & Life Sciences, Chonnam National University, Gwangju 61186, Republic of Korea; hblee@chonnam.ac.kr

**Keywords:** ectomycorrhizal fungi, comparative genomics, gene flow, niche differentiation

## Abstract

Ecological speciation via divergent selection despite gene flow remains poorly documented in fungi. We integrated niche modeling and comparative genomics to examine ecological and genomic divergence among three ectomycorrhizal *Thelephora* species. *T. ganbajun* is restricted to mid-elevation Yunnan with climatically stable environments; *T. aurantiotincta* occupies lower elevations with pronounced temperature seasonality; and *T. terrestris* displays the broadest distribution, tolerating extreme temperature fluctuations and low precipitation. Phylogenomic analysis recovered the three species as distinct lineages, with *T. terrestris* diverging at 21.16 Mya and *T. ganbajun* and *T. aurantiotincta* splitting at 14.97 Mya. Gene flow analysis revealed unidirectional introgression: *T. terrestris* donated to both other species, and *T. aurantiotincta* donated to *T. ganbajun*, with negligible reverse migration. *T. terrestris* possesses the largest genome (40.73 Mb) with recent LTR retrotransposon expansion and amplified cytochrome P450 and lignocellulose-active CAZymes. *T. ganbajun* shows expanded copper transport and cell wall remodeling genes, while *T. aurantiotincta* exhibits expansion of fatty acid metabolism genes. These results document pronounced niche differentiation, genomic divergence, and directional historical gene flow among closely related species, providing an ecological and genomic basis for examining ecological speciation in fungi.

## 1. Introduction

Niche differentiation is a fundamental mechanism driving speciation and maintaining biodiversity. Modern coexistence theory indicates that stable species coexistence depends on the balance between the degree of niche differentiation and differences in competitive ability [1]. For closely related species, niche differentiation can be achieved through multiple pathways. Differentiation through resource utilization and microhabitat selection reduces direct interspecific competition, facilitating multi-species coexistence at local scales [2,3]. In contrast, adaptive differentiation in response to environmental factors such as temperature and moisture shapes species distribution patterns at regional and continental scales. These two types of niche differentiation often act in concert to determine species’ geographic distributions and community composition. Although niche differentiation has been extensively studied in animals and plants, research on fungi, particularly ectomycorrhizal (ECM) fungi, remains relatively limited [4,5,6,7]. Nevertheless, ECM fungi have recently emerged as ideal model systems for studying niche theory due to their unique ecological attributes [8]. Niche differentiation among ECM fungi is achieved through multiple pathways, with host selection and environmental adaptation serving as key mechanisms. At the host selection level, host specificity ranges from strict specialization to flexible generalism. The genus *Suillus* exhibits host specificity toward particular Pinaceae taxa, with different subgenera specialized on Larix, Pinus subgenus Strobus, or Pinus subgenus Pinus, and shifts in host specificity have driven species diversification within *Suillus* [9]. At the environmental adaptation level, continental- and global-scale studies reveal that ECM fungi exhibit biogeographic patterns primarily driven by climate, possess narrower climatic niches compared to saprotrophic fungi, and display pronounced species differentiation along temperature and precipitation gradients [10]. These distinct niche differentiation patterns reflect fungal responses to different selective pressures. During adaptation to new ecological niches, fungi typically undergo genomic remodeling, including expansion and contraction of gene families, changes in non-coding elements, and genomic structural variations. With advances in genome sequencing and analytical technologies, niche differentiation can now be dissected at the molecular level, revealing the genetic basis underlying adaptive species divergence.

Comparative genomics and population genomics provide powerful tools for revealing the genetic basis of ecological niche differentiation in ECM fungi. Comparative genomic studies have revealed that genome remodeling in ECM fungi is closely associated with their ecological adaptation. Research on the genus *Suillus* has demonstrated highly dynamic genomes, with expansion of secondary metabolite biosynthesis genes and oxidation-reduction-related genes enabling adaptation to the chemical environment and reactive oxygen species stress of different hosts [11]. Different lineages within Boletales retain differentiated combinations of plant cell wall-degrading enzymes, and their varying lignocellulose decomposition capacities may be related to soil organic matter acquisition [12]. Population genomic studies have also proven to be powerful in elucidating intraspecific differentiation mechanisms in ECM fungi. Population genomic analysis of Boletus species revealed that environment-driven genomic differentiation is maintained despite ongoing gene introgression [13]. Landscape genomics approaches, including Redundancy Analysis (RDA), Latent Factor Mixed Models (LFMM), and Gradient Forest machine learning, can identify adaptation-related genes and predict distribution patterns under climate change by integrating genomic data with geospatial climate data [14]. Additionally, ecological niche models provide complementary tools for predicting the geographic distribution of ECM fungi, with climatic factors considered key drivers of distribution patterns [15].

The genus *Thelephora* is widely distributed across temperate to tropical regions globally and represents one of the important ectomycorrhizal fungal groups [16,17]. *Thelephora ganbajun*, *T. aurantiotincta*, and *T. terrestris* are three closely related species that exhibit pronounced ecological niche differentiation in China. *T. ganbajun* is primarily concentrated in Yunnan Province, southwestern China, with a limited distribution range, and primarily forms ectomycorrhizal associations with *Pinus yunnanensis* [18,19,20]; *T. terrestris* displays cosmopolitan distribution characteristics, occurring from subtropical to cold-temperate zones and adapting to diverse environmental conditions, and is a broad-host ectomycorrhizal fungus capable of forming symbiotic relationships with a wide range of coniferous and broadleaf tree species [21,22]; and *T. aurantiotincta* exhibits intermediate ecological adaptability, mainly distributed in central and southern China, and mainly forms mycorrhizal associations with trees of Fagaceae and Pinaceae [23]. Geographically, *T. ganbajun* and *T. aurantiotincta* are peripatrically distributed in southwestern China, whereas both species occur parapatrically with *T. terrestris*. Meanwhile, as closely related taxa, the three species possess comparable phylogenetic relationships, with geographic distributions showing clear separation yet varying degrees of overlap, providing a rare opportunity to study gene flow and ecological niche differentiation [22]. Furthermore, these three species are all important edible and medicinal fungal resources whose populations face threats from over-collection and habitat loss, making research on their ecological adaptation mechanisms of conservation importance [24,25,26].

This study investigates three closely related species, *T. ganbajun*, *T. aurantiotincta*, and *T. terrestris*, using an integrated approach combining ecological niche modeling and comparative genomics to describe their ecological niche differences and genomic divergence. The main research objectives include: (1) predicting the suitable habitat distribution of the three species in China and quantifying their ecological niche characteristics and geographic overlap patterns; (2) resolving the phylogenetic relationships, divergence times, and genome architectural features of the three species, including the spatial distribution patterns of transposable elements and their relationships with different functional gene categories; (3) characterizing patterns of gene family expansion and contraction in the three species through functional enrichment analysis of expanded gene families, including comparative analysis of carbohydrate-active enzyme (CAZyme) families, and identifying candidate functional hypotheses; and (4) describing historical genetic exchange among the three species through gene flow analysis. The genomic differences among the three species described below may also reflect neutral evolutionary processes or historical contingencies unrelated to their current environments, rather than adaptation. We note that our comparative genomic analyses are based on a single genome per species; the functional interpretations presented here are therefore advanced as hypotheses to be tested rather than as demonstrated species-level adaptive strategies. This study aims to improve our understanding of ecological niche differentiation and genomic divergence among closely related ectomycorrhizal fungi, and to provide an ecological and genomic basis for examining the role of ecological selection in their divergence.

## 2. Materials and Methods

### 2.1. Ecological Niche Analysis

Based on field surveys conducted from 2022 to 2024, we recorded fruiting body distribution points for three *Thelephora* species: *T. ganbajun* (87 points), *T. terrestris* (45 points), and *T. aurantiotincta* (38 points). To avoid overfitting caused by sampling bias from localized distribution points, spatial filtering was performed using ArcGIS 10.2.1 with the WorldClim 5 arc-minute grid (~10 km) to match the resolution of the environmental variables [27]. Only one occurrence point was retained per grid cell to reduce spatial autocorrelation and sampling bias. After spatial filtering, 70, 30, and 18 valid distribution points were retained for *T. ganbajun*, *T. terrestris*, and *T. aurantiotincta*, respectively, for subsequent modeling. Current-period bioclimatic variables and elevation data were obtained from the WorldClim database at a spatial resolution of 5 arc-minutes [28], with all raster-format environmental variables standardized to the WGS84 geographic coordinate system. To avoid model overfitting, a Pearson correlation matrix was computed among 20 candidate variables using SPSS 26.0 [29]. Variables with |r| ≥ 0.8 were iteratively removed, and six weakly correlated variables were retained for modeling: annual mean temperature (Bio1), mean diurnal range (Bio2), isothermality (Bio3), annual precipitation (Bio12), precipitation seasonality (Bio15), and elevation.

An ensemble modeling approach was employed using the Biomod2 package (v4.3.1) in R (4.3.2) to predict the ecological niches of the three species [30]. Model performance was assessed using spatially structured cross-validation [31]. A total of 1000 pseudo-absence points were randomly generated within the study area. Ensemble models were constructed using the median probability across all single models (EMmedian). Model accuracy was evaluated using AUC, TSS, and Kappa values, which were 0.998, 0.988, and 0.880 for *T. aurantiotincta*; 1.000, 0.991, and 0.970 for *T. ganbajun*; and 0.978, 0.902, and 0.710 for *T. terrestris*, respectively [32].

To incorporate host vegetation constraints, the 1:1,000,000 vegetation type map of China was used to generate a forest mask retaining only coniferous forest, broadleaf forest, and coniferous–broadleaf mixed forest vegetation-type groups compatible with an ectomycorrhizal habit. All non-forest vegetation types (shrubland, meadow, steppe, grass-tussock, marsh, desert, alpine vegetation, cultivated vegetation, and non-vegetated areas) were excluded. The forest vegetation polygons were reprojected to match the prediction raster coordinate system and rasterized, and the forest mask was applied band-by-band to the biomod2 ensemble predictions, setting the suitability probability to zero in all non-forest areas. All maps were reprojected to the Albers equal-area conic projection suitable for China, and the standard base map approved by the Ministry of Natural Resources of China (approval number: GS (2024) 0650) was used, with the South China Sea shown in a standardized inset.

### 2.2. Gene Prediction and Functional Annotation

The genome of *T. ganbajun* was newly sequenced in this study. Genomic DNA was extracted from fruiting body tissue and sequenced using PacBio HiFi technology to generate high-fidelity reads with accuracy exceeding 99.9%. De novo assembly was performed using hifiasm, and assembly quality was assessed using BUSCO 5.8.2. The assembly has been deposited in the figshare database (DOI: https://doi.org/10.6084/m9.figshare.32593176). The genome assemblies of *T. aurantiotincta* MG58 (GCA_003316405.1) [33], *T. terrestris* UH-Tt-Lm1 (GCA_015956445.1) [16], *Sarcodon aspratus* MG57 (GCA_003313825.1) [33], *Ganoderma boninense* G3 (GCA_002900995.3) [34], *G. leucocontextum* Dai12418 (GCA_022813035.1) [35], *G. sinense* ZZ0214-1 (GCA_002760635.1) [36], *Rhizopogon salebrosus* TDB-379 (GCA_029603245.1), and *R. vesiculosus* AM-OR11-056 (GCA_001882365.1) were retrieved from the NCBI GenBank database. The *T. ganbajun* assembly comprises 200 contigs with a total length of 29.41 Mb and a contig N50 of 3.62 Mb. The *T. terrestris* assembly comprises 76 contigs, 40.7 Mb total length, and contig N50 of 2.1 Mb. The *T. aurantiotincta* assembly comprises 9339 scaffolds, 29.4 Mb total length, and scaffold N50 of 10.2 kb. The genome assemblies of *Phellinus viticola* (PhevitSig-SM15 v1.0) and *P. igniarius* (CCBS 575 v1.0) were obtained from the (JGI MycoCosm portal https://mycocosm.jgi.doe.gov, accessed on 5 October 2025).

Gene prediction and functional annotation were performed on the genomes of *T. ganbajun*, *T. terrestris*, and *T. aurantiotincta*. For all other species, gene annotations were obtained directly from their respective database submissions. For the three *Thelephora* species, gene prediction combined ab initio and homology-based approaches. Ab initio gene prediction was conducted using AUGUSTUS v3.5.0 [37]. Homology-based gene prediction utilized Exonerate v2.2.0, GeneWise v2.4.1, and GenomeThreader v1.7.3 to align protein sequences from closely related species to repeat-masked genomes [38,39]. All prediction results were integrated using EvidenceModeler v1.1.1, and gene models were filtered through protein domain identification using HMMER v3.3.2 via the Pfam database and BLASTP searches using DIAMOND v2.1.8 [40,41,42,43]. Functional annotation of predicted coding sequences was performed through BLASTP searches against multiple databases, including the NCBI non-redundant protein database, Swiss-Prot, InterPro, Pfam, eggNOG, TMHMM, and PredGPI. Coding sequences were aligned to the KOG database with parameters of coverage > 20% and identity > 30% [44,45]. Carbohydrate-active enzymes were annotated using dbCAN [46]. Assembly completeness for the three *Thelephora* genomes was assessed using BUSCO 5.8.2 with the basidiomycota_odb12 lineage dataset.

### 2.3. Phylogenetic Tree Construction

The phylogenetic tree was constructed to provide a dated framework for divergence time estimation and gene family expansion/contraction analysis of the three target *Thelephora* species, rather than to reconstruct a large-scale phylogeny of Basidiomycota. Phylogenetic tree construction was performed using *T. ganbajun*, *T. aurantiotincta*, and *T. terrestris* from the genus *Thelephora*, along with *Sarcodon aspratus*, a closely related genus within *Thelephorales*, to provide phylogenetic context. Additionally, five species from related orders were included, namely *Ganoderma boninense*, *G. leucocontextum*, *G. sinense*, and two *Boletales* species, *Rhizopogon salebrosus* and *R. vesiculosus*. *Phellinus viticola* and *P. igniarius* were used as outgroups. These taxa were selected at moderate phylogenetic distances from Thelephorales to enable precise placement of the three fossil calibration points described below. Genome assemblies and gene structure annotation files for all species were obtained as described above. Data preprocessing included CDS sequence correction, gene ID standardization, and extraction of protein and CDS sequences. Based on the genomic data, genome-wide protein sequence alignment was first performed using Diamond software with an E-value threshold of ≤1 × 10^−5^, a minimum sequence identity of 10%, and a maximum of 50 target sequences retained per query sequence [47]. The alignment results were stringently filtered (E-value ≤ 1 × 10^−9^, both query and target coverage ≥ 50%, conservative pairwise ratio ≥ 30%), and orthologous gene families were identified through clustering using the MCL algorithm implemented in OrthoFinder v2.2 [48]. Single-copy orthologous genes present in all species were extracted from the clustering results (requiring at least 50% of species to have single-copy genes, maximum of 10 copies per gene family, sequence length ≤ 6000 bp). Multiple sequence alignment of protein sequences was performed using the L-INS-i algorithm in MAFFT v7.407 [49], followed by codon-guided alignment of CDS sequences based on the protein alignment. Gblocks software (version 0.91b) was used to extract conserved blocks and remove low-quality and highly variable regions [50]. The conserved regions from all gene families were concatenated into a gene matrix, converted to Phylip format, and used to construct a maximum likelihood phylogenetic tree using RAxML v8.2.12 with the GTRGAMMA model and 1000 bootstrap replicates. Species divergence times were estimated using the MCMCTree program in PAML v4.9i [51,52], employing the Markov chain Monte Carlo (MCMC) method with three fossil calibration points: Ganodermites lybicus to calibrate the ancestral node of Polyporales (18–50 Mya), Quatsinoporites cranhamii to calibrate the ancestral node of Hymenochaetales (127–250 Mya), and the Rhizopogon ECM ancestral node (50–100 Mya) [53]. These three calibration points were selected from the default calibration scheme described above, which includes eight fossils confirmed to be conflict-free through fossil cross-validation. These calibration points span from 18–50 Mya to 127–250 Mya, covering different hierarchical levels of the phylogenetic tree and providing multi-level temporal constraints for divergence time estimation near the target nodes. We note that the Agaricomycetes fossil record is sparse, that fossil-based calibration points represent minimum-age constraints rather than direct estimates of node age, and that the resulting divergence time estimates should be interpreted as approximate.

### 2.4. Gene Family Expansion and Contraction Analysis

Based on the orthologous gene clustering results from OrthoFinder, gene families present in at least 40% of species were extracted, without restrictions on single-copy gene proportion or copy number upper limits. Genes not assigned to any orthogroup containing a sequence from another taxon in the eleven-genome OrthoFinder analysis are referred to as lineage-restricted genes. Due to the potential impact of gene family size heterogeneity on the analytical accuracy of CAFE5 (version 5.1), gene families were filtered according to maximum fold-change threshold (10-fold) and maximum gene family size, dividing the data into normal and outlier groups for separate processing. The normal data group was analyzed using CAFE5 software to estimate the error model for correcting gene count statistical errors, while simultaneously optimizing the lambda value [54]. Lambda values, error model parameters, and the maximum possible lambda value under the tree topology were extracted from the normal group analysis results. These estimated parameters were then fixed and applied to an independent analysis of the outlier data group, ensuring consistent evolutionary rate parameters across both datasets. The results files from both analyses were integrated, including information on gene family number changes across different branches for each species and branch probability information. Based on conditional probability (*p* < 0.05), significant expansion or contraction events of gene families on specific branches were determined, and the number of significantly changed gene families on each branch was quantified.

### 2.5. Positive Selection and Rapidly Evolving Gene Analysis

Orthologous genes present in at least 60% of the species were extracted from the OrthoFinder clustering results. Protein sequences were aligned using the L-INS-i algorithm in MAFFT software, and the alignments were subsequently converted to corresponding CDS sequence alignments. For identification of positively selected genes (PSGs), preliminary screening was conducted using the yn00 program and the free-ratio model (model = 1) of the codeml program in PAML, retaining candidate genes with ω > 1.0 [52,55]. Subsequently, these candidate genes were analyzed using the branch-site model (model = 2, NSsites = 2) in the codeml program, which allows ω > 1 at specific sites along foreground branches [56]. Likelihood ratio tests (LRT) were performed to compare the alternative model (allowing positive selection) with the null model (not allowing positive selection), combined with the Bayes Empirical Bayes (BEB) method to identify specific positively selected sites [57]. Genes with *p*-value < 0.05 and significant positively selected sites detected by BEB analysis were identified as positively selected genes.

For identification of rapidly evolving genes (REGs), Gblocks software (version 0.91b) was used to extract conserved blocks from CDS alignments, removing low-quality and highly variable regions. Subsequently, hierarchical analysis was performed using the codeml program in PAML software: the free-ratio model (model = 1) estimated independent ω values for each branch, the one-ratio model (model = 0) estimated the average ω value across the entire tree, and the two-ratio model (model = 2) estimated ω values separately for the designated foreground and background branches [50]. For each target species as the foreground branch, likelihood ratio tests were performed to compare the two-ratio model with the one-ratio model, followed by FDR multiple testing correction [58]. Genes satisfying three conditions simultaneously were identified as rapidly evolving genes for that species: the foreground branch ω value was significantly greater than the tree-wide average ω value, the foreground branch ω value was significantly greater than the background branch ω value, and the FDR-corrected *p*-value < 0.05.

### 2.6. Repeat Sequence Analysis

Repeat sequence analysis of the three *Thelephora* species was performed using a combination of database-based masking and de novo prediction approaches. Initial repeat sequence masking was conducted using RepeatMasker v4.1.5 combined with the RepBase fungal repeat sequence library [59,60]. De novo repeat sequence identification was performed using RepeatModeler v2.0.5 to construct custom repeat sequence libraries, including database construction and LTR structure analysis. Subsequently, comprehensive transposable element annotation was performed using EDTA v2.0.1, and RepeatMasker v4.1.5 was run separately using custom libraries generated by RepeatModeler and EDTA to perform additional genome masking [61,62]. All RepeatMasker output files were merged using the merge_repeatMasker_out.pl script to generate comprehensive statistics. LTR insertion times were estimated using LTR_FINDER, with the fungal mutation rate set to 1.05 × 10^−9^ [63]. LTR sequences from the three *Thelephora* species were aligned using MAFFT v7.407, and a phylogenetic tree was constructed using IQ-TREE 2 [49,64]. Distances between TEs and coding genes were calculated using different pipeline scripts, and statistical significance of the results was tested using Kruskal–Wallis and Dunn’s tests [65,66]. For each gene, the distance to the nearest transposable element was measured in base pairs between the nearest boundaries of the two features, with a distance of 0 bp indicating overlap. Distances were not measured across contig boundaries. No distance threshold was used to define ‘adjacent’; rather, the distance to the nearest transposable element was directly measured for every gene, and these distances were then grouped by gene category for comparison.

### 2.7. Gene Flow Analysis

Gene flow between species was evaluated using the 3s software based on the isolation-with-migration model [67]. Single-copy orthologous genes present in all species, appearing in at least 60% of species, and with sequence length not exceeding 2000 bp were extracted from the OrthoFinder clustering results. Protein sequences were aligned using the L-INS-i algorithm in MAFFT software, and the alignments were converted to corresponding CDS sequence alignments. The alignment results were converted to Phylip format and merged into a single input file. In the triplet analysis, it was assumed that species 3 (the outgroup) does not exchange genes with species 1 and 2. *Ganoderma leucocontextum* (Polyporales) was selected as the outgroup because it is at a moderate phylogenetic distance from the ingroup and, as a member of a different order, can reasonably be assumed to have no gene flow with the three *Thelephora* species. Species grouping files (Imap) and control files were configured according to phylogenetic relationships, and target species were divided into multiple triplet combinations for analysis. By comparing gene flow parameters between different species pairs, historical gene exchange events and their directionality were inferred. Gene flow analysis was performed using the isolation-with-migration model. The parameter τ represents the species divergence time (measured in units of expected mutations per site), θ represents the population size parameter (θ = 4 Nμ, where N is the effective population size and μ is the mutation rate per site per generation), and M represents the migration rate (M = Nm, where m is the probability of migration per individual per generation). These parameters describe the historical pattern of divergence and genetic exchange between species pairs.

## 3. Results

### 3.1. Geographic Distribution and Ecological Niche Differentiation

**Figure 1:**
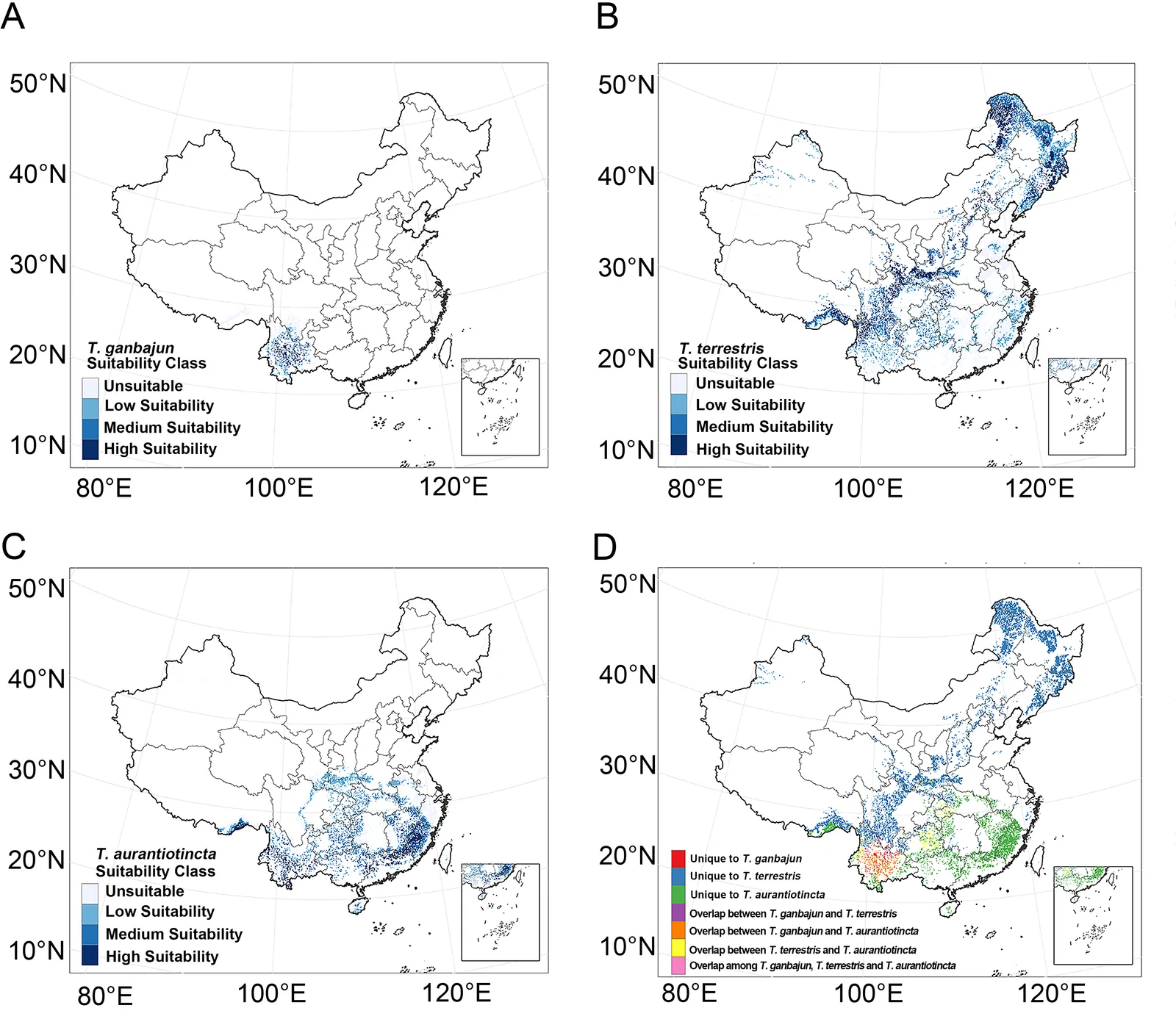
Habitat suitability distribution of three *Thelephora* species in China. (**A**) Distribution of suitable habitats for *T. ganbajun*. (**B**) Distribution of suitable habitats for *T. aurantiotincta*. (**C**) Distribution of suitable habitats for *T. terrestris*. (**D**) Spatial overlap relationships of the three species’ distribution ranges, with different colors representing species-specific distributions and co-distribution areas.

Species distribution models predicted that under current climatic conditions, the suitable habitat for *T. ganbajun* is primarily concentrated in southwestern regions of Yunnan Province (284,817 km^2^, accounting for 79.2%) and Sichuan Province (39,502 km^2^), with the total area of high and moderate suitability zones reaching 359,442 km^2^ (Figure 1A). The suitable habitat for *T. aurantiotincta* extends beyond Yunnan and Sichuan to include extensive distributions across southern and southwestern provinces such as Guizhou, Jiangxi, Hunan, Guangxi, and Fujian, with medium to high suitability zones totaling 1,466,096 km^2^ (Figure 1B). *T. terrestris* exhibits the most extensive distribution range, occurring not only in southwestern China but also widely distributed in northern and high-altitude regions including Inner Mongolia (543,551 km^2^), Xizang (377,523 km^2^), Heilongjiang (357,309 km^2^), and Qinghai (257,205 km^2^), with highly suitable areas covering 3,216,916 km^2^ (Figure 1C). The distribution ranges of the three species show varying degrees of overlap (Figure 1D). The common suitable area shared by all three species is 71,001 km^2^, primarily distributed in Yunnan Province (29,858 km^2^), Guizhou Province (17,760 km^2^), Sichuan Province (16,439 km^2^), and southeastern Xizang (9795 km^2^). Among pairwise overlaps, the co-distribution area of *T. terrestris* and *T. aurantiotincta* is the largest (343,985 km^2^), mainly located in Guizhou, Sichuan, Hubei, and Chongqing; the co-distribution area of *T. ganbajun* and *T. aurantiotincta* is intermediate (197,773 km^2^), concentrated in Yunnan; and the co-distribution area of *T. ganbajun* and *T. terrestris* is the smallest (10,286 km^2^), restricted to localized regions in Yunnan and Sichuan. The unique distribution area of *T. terrestris* is primarily located in northeastern, northern, and northwestern China. The unique distribution area of *T. aurantiotincta* is concentrated in central and southern China, while the unique distribution area of *T. ganbajun* is almost entirely confined to Yunnan Province. This distribution pattern indicates that *T. ganbajun* likely has strict requirements for specific environmental conditions; *T. terrestris* demonstrates broad ecological adaptability, capable of adapting to diverse environments from high-altitude subtropical to cold-temperate zones; and *T. aurantiotincta* exhibits intermediate ecological adaptability, primarily occupying central and southern China.

The differential suitable habitats of the three *Thelephora* species reflect their distinct ecological niche characteristics (Table 1). To minimize prediction errors at the margins of species distribution models and exclude extreme outliers, we extracted environmental variable ranges using the 10th to 90th percentile interval, thereby retaining the central 80% of the data within moderate-to-high suitability areas. In terms of temperature, *T. ganbajun* shows a clear preference for warm climatic conditions, with a median annual mean temperature of 15.17 °C (range: 11.19–19.11 °C) and a median minimum temperature of the coldest month of 1.67 °C (range: −2.66–5.64 °C), indicating that it is mainly distributed in regions without severe cold. *T. terrestris* is adapted to colder environments, with a median annual mean temperature of only 4.13 °C (range: −3.32–15.05 °C) and minimum temperature in the coldest month reaching as low as −28.19 °C, demonstrating adaptability to cold-temperate and high-latitude regions. The temperature adaptation range of *T. aurantiotincta* is intermediate, with a median annual mean temperature of 15.79 °C (range: 11.01–19.11 °C), but its temperature seasonality (Bio4 median of 760) is significantly higher than that of *T. ganbajun* (452), indicating its ability to tolerate greater seasonal temperature variations. Regarding precipitation, the potential distribution area of *T. ganbajun* possesses relatively stable precipitation conditions, with a median annual precipitation of 1008 mm (range: 812–1458 mm), median precipitation of the driest period of 12 mm, and median coefficient of variation in precipitation seasonality of 84.22. *T. aurantiotincta* exhibits a broad adaptation range to precipitation, with a median annual precipitation of 1201 mm (range: 787–1652 mm) and precipitation during the driest period of 8–43 mm, indicating its preference for relatively humid environments. *T. terrestris* is mainly distributed in regions with lower precipitation, with a median annual precipitation of only 564 mm (range: 332–1079 mm) and median precipitation of the driest period of 4 mm (range: 2–16 mm), demonstrating higher tolerance to arid environments. Elevation distribution characteristics show that *T. ganbajun* is primarily distributed in mid-elevation mountainous areas (median 1908 m, range 1360–2514 m). *T. terrestris* exhibits a wide elevation span (median 1039 m, range 122–4451 m). *T. aurantiotincta* has the lowest median elevation (461 m, range 32–2058 m), primarily occupying low- to mid-elevation environments.

### 3.2. Phylogenomic Divergence and Genome Architecture

**Figure 2:**
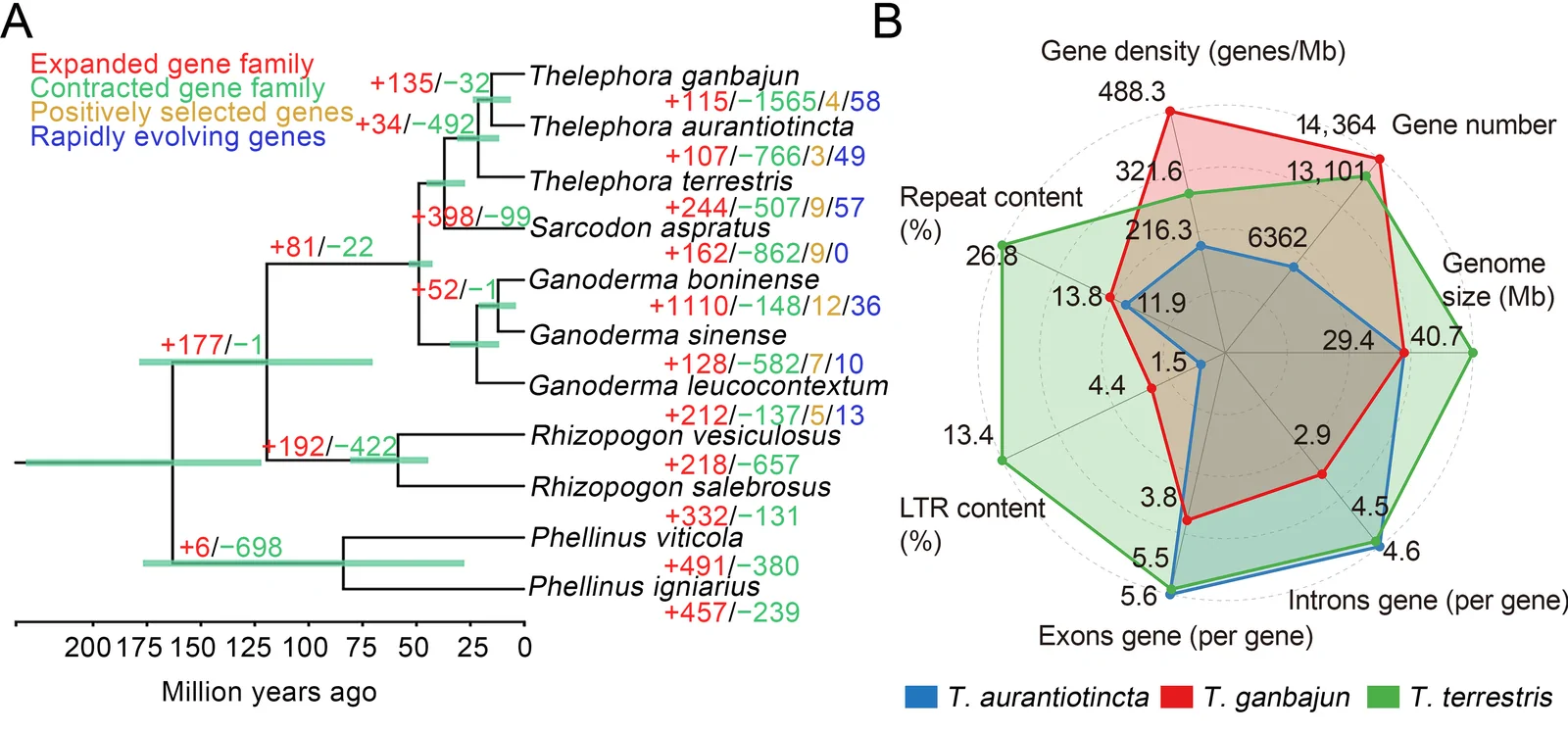
Phylogenetic relationships and genomic features of three *Thelephora* species. (**A**) Phylogenetic tree based on whole-genome data showing relationships among three *Thelephora* species and other Agaricomycetes. Numbers on branches indicate expanded/contracted gene families, positively selected genes, and rapidly evolving genes. Time scale in million years. (**B**) Genomic feature comparison. Radar plot displays genome size (Mb), gene number, gene density (genes/Mb), repeat content (%), LTR content (%), and exons per gene.

Phylogenetic trees constructed from genomic data revealed that three *Thelephora* species formed distinct lineages and exhibited a sister-group relationship with the genus *Sarcodon* (Figure 2A). Molecular clock analysis indicated that *T. terrestris* diverged first at 21.16 Mya (95% HPD: 11.41–30.97 Mya), followed by the divergence of *T. ganbajun* and *T. aurantiotincta* at 14.97 Mya (95% HPD: 6.02–23.66 Mya).

Comparative genomic analysis revealed structural differences among the three species (Figure 2B). *T. terrestris* possessed the largest genome (40.73 Mb), larger than *T. ganbajun* and *T. aurantiotincta*, primarily attributed to transposable element proliferation. The repeat content of *T. terrestris* reached 26.79%, with its LTR content (13.41%) being threefold higher than *T. ganbajun* (4.43%) and *T. aurantiotincta* (1.46%), indicating that LTR retrotransposon expansion was the primary driver of genome enlargement in *T. terrestris*. Gene number also varied among the three species. *T. ganbajun* exhibited the highest gene count (14,364) and gene density (488.3 genes/Mb), whereas *T. aurantiotincta* had the fewest genes (6362) and the lowest gene density (216.3 genes/Mb). BUSCO completeness was 97.7% for *T. terrestris*, 90.2% for *T. ganbajun*, and 77.1% for *T. aurantiotincta*, indicating that the three assemblies differ in completeness; the low gene count of *T. aurantiotincta* is therefore likely to reflect assembly incompleteness in part rather than gene content alone. Gene structure analysis showed that *T. aurantiotincta* and *T. terrestris* averaged 5.58 and 5.46 exons per gene, respectively, while *T. ganbajun* contained only 3.87 exons per gene.

### 3.3. Transposable Element Analysis

**Figure 3:**
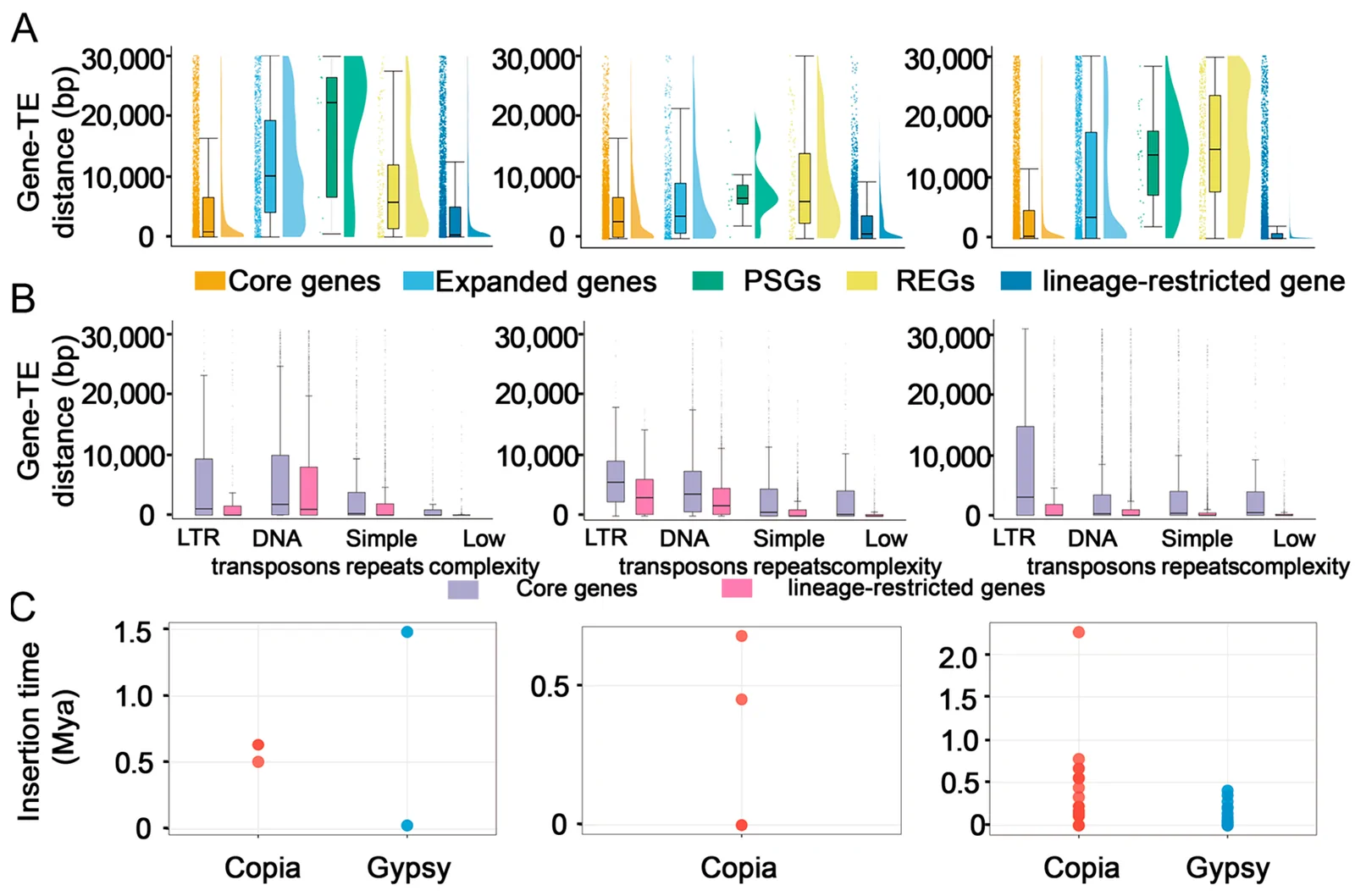
Spatial distribution of transposable elements and genes, and LTR insertion time analysis. (**A**) Distance distribution between five gene categories (core genes, expanded genes, positively selected genes, rapidly evolving genes, and lineage-restricted genes) and nearest transposable elements (0–30 kb). (**B**) Distance distribution between four transposable element types (LTR retrotransposons, DNA transposons, simple repeats, and low-complexity sequences) and core genes versus lineage-restricted genes. (**C**) Insertion time distribution of Copia and Gypsy LTR retrotransposons.

To investigate the impact of transposable elements on genome structure and gene functional divergence, we analyzed the spatial distance distribution between transposable elements and different gene types in the three *Thelephora* species and estimated the insertion times of LTR retrotransposons. Differences existed in the spatial relationships between different gene categories and transposable elements (Figure 3A). Core genes maintained relatively greater distances from transposable elements, with median distances of 6255 bp in *T. ganbajun*, 3960.5 bp in *T. aurantiotincta*, and 5678 bp in *T. terrestris*. Lineage-restricted genes exhibited high proximity to transposable elements, with 50.66–55.74% of transposable elements located near lineage-restricted genes, and median distances of 4253 bp in *T. ganbajun*, 2077 bp in *T. aurantiotincta*, and only 955.5 bp in *T. terrestris*. In *T. aurantiotincta*, only 5.37% of transposable elements (571) were adjacent to expanded genes, with a median distance of 4852 bp, whereas in *T. ganbajun*, 35.21% of transposable elements (3325) were adjacent to expanded genes, but with an extremely large median distance (187,916.5 bp), indicating that expanded genes are often located in transposable element-sparse genomic regions. Positively selected genes (PSGs) and rapidly evolving genes (REGs) showed the most distant relationships with transposable elements. LTR retrotransposons among transposable elements exhibited the strongest gene category selectivity (Figure 3B). In *T. terrestris*, the median distance between core genes and LTRs was 2925 bp, while the median distance for lineage-restricted genes was 0 bp, revealing extreme enrichment of lineage-restricted genes in LTR-dense regions. *T. aurantiotincta* presented a similar pattern, with median distances of 5501 bp for core genes and 2978 bp for lineage-restricted genes from LTRs. In *T. ganbajun*, the median distance between core genes and LTRs was 1007.5 bp, while the median distance for lineage-restricted genes was 0 bp. LTR retrotransposon insertion time analysis revealed that the three species experienced different LTR expansion histories (Figure 3C). *T. terrestris* possessed abundant LTR data, displaying a clear recent expansion pattern. Copia-type LTR insertion times were mainly concentrated within 0.5 million years, with only a few traceable to 2.26 million years ago. Gypsy-type LTR insertion times were even more concentrated, with almost all occurring within 0.4 million years, indicating that this type underwent extremely recent rapid expansion. In contrast, *T. ganbajun* and *T. aurantiotincta* had fewer intact LTRs detected, limiting detailed statistical analysis. Copia-type LTR insertion times in *T. ganbajun* were concentrated at 0.5–0.7 million years ago, while its Gypsy-type LTRs showed one extremely recent insertion and one more ancient insertion. The Copia-type LTRs in *T. aurantiotincta* had insertion times spanning 0–0.68 million years. These analyses indicate that transposable element distribution is closely correlated with gene functional categories. Lineage-restricted genes are significantly enriched in transposable element-dense regions, especially near LTR retrotransposons, while core genes, positively selected genes, and rapidly evolving genes tend to be distributed in transposable element-sparse genomic regions. The recent large-scale LTR expansion in *T. terrestris* is consistent with its higher LTR content (13.41%) and largest genome size (40.73 Mb). In this species, lineage-restricted genes are located significantly closer to LTR retrotransposons than core genes are.

### 3.4. Gene Family Analysis

Gene family evolution analysis revealed that *T. ganbajun* experienced expansion of 115 gene families and contraction of 1565 gene families. Additionally, 4 positively selected genes and 58 rapidly evolving genes were detected in this species. *T. aurantiotincta* possessed only 107 expanded gene families but a high number of contracted gene families (766), along with 3 positively selected genes and 49 rapidly evolving genes. *T. terrestris* harbored 244 expanded gene families and 507 contracted gene families, while possessing 9 positively selected genes and 57 rapidly evolving genes.

Expanded gene families in *T. ganbajun* were enriched in copper ion transport and regulation functions, including P-type divalent copper transporter activity (GO:0043682, *p* = 1.65 × 10^−7^), copper ion homeostasis (GO:0055070, *p* = 1.65 × 10^−7^), and monoatomic cation transport (GO:0006812, *p* = 2.17 × 10^−5^). Expanded CAZyme families in *T. ganbajun* included glycoside hydrolase family 23 (GH23), glycosyltransferase family 2 (GT2), and carbohydrate-binding module 50 (CBM50), enzyme classes active on microbial cell wall polysaccharides such as peptidoglycan and chitin.

Expanded and rapidly evolving genes in *T. terrestris* exhibited functional enrichment patterns related to environmental adaptation. Cytochrome P450 system-related genes showed expansion, including heme binding (GO:0020037, *p* = 3.97 × 10^−13^), iron ion binding (GO:0005506, *p* = 1.38 × 10^−12^), monooxygenase activity (GO:0004497, *p* = 1.06 × 10^−11^), and oxidoreductase activity (GO:0016705, *p* = 1.72 × 10^−11^). Protein phosphorylation signaling system-related genes also exhibited expansion, including protein phosphorylation (GO:0006468, *p* = 3.33 × 10^−9^) and protein serine/threonine kinase activity (GO:0004674, *p* = 1.80 × 10^−9^). Rapidly evolving genes were enriched in autophagosome assembly (GO:0000045, *p* = 1.13 × 10^−4^) and cellular response to starvation (GO:0009267, *p* = 3.94 × 10^−4^). Positively selected genes included Hsp20, a heat shock protein, and DnaJ, a molecular chaperone. Expanded CAZyme families in *T. terrestris* included carbohydrate-binding module 13 (CBM13), carbohydrate esterase family 5 (CE5), and auxiliary activity family 9 (AA9), enzyme classes active on plant cell wall polysaccharides.

Expanded genes in *T. aurantiotincta* were enriched in fatty acid metabolism-related functions, including medium-chain fatty acid-CoA ligase activity (GO:0031956, *p* = 2.80 × 10^−12^), FAD binding (GO:0071949, *p* = 5.10 × 10^−9^), methylation (GO:0032259, *p* = 6.88 × 10^−11^), and phosphatidylcholine biosynthesis (GO:0006656, *p* = 5.74 × 10^−6^).

### 3.5. Gene Flow Analysis

To detect gene flow among the three *Thelephora* species, likelihood ratio tests were performed using *Ganoderma leucocontextum* as an outgroup. All tests significantly rejected the null hypothesis of no gene flow (2ΔlnL = 30.72–66.51, *p* < 0.05), supporting the symmetric isolation-with-migration model and confirming historical gene flow among these species.

**Figure 4:**
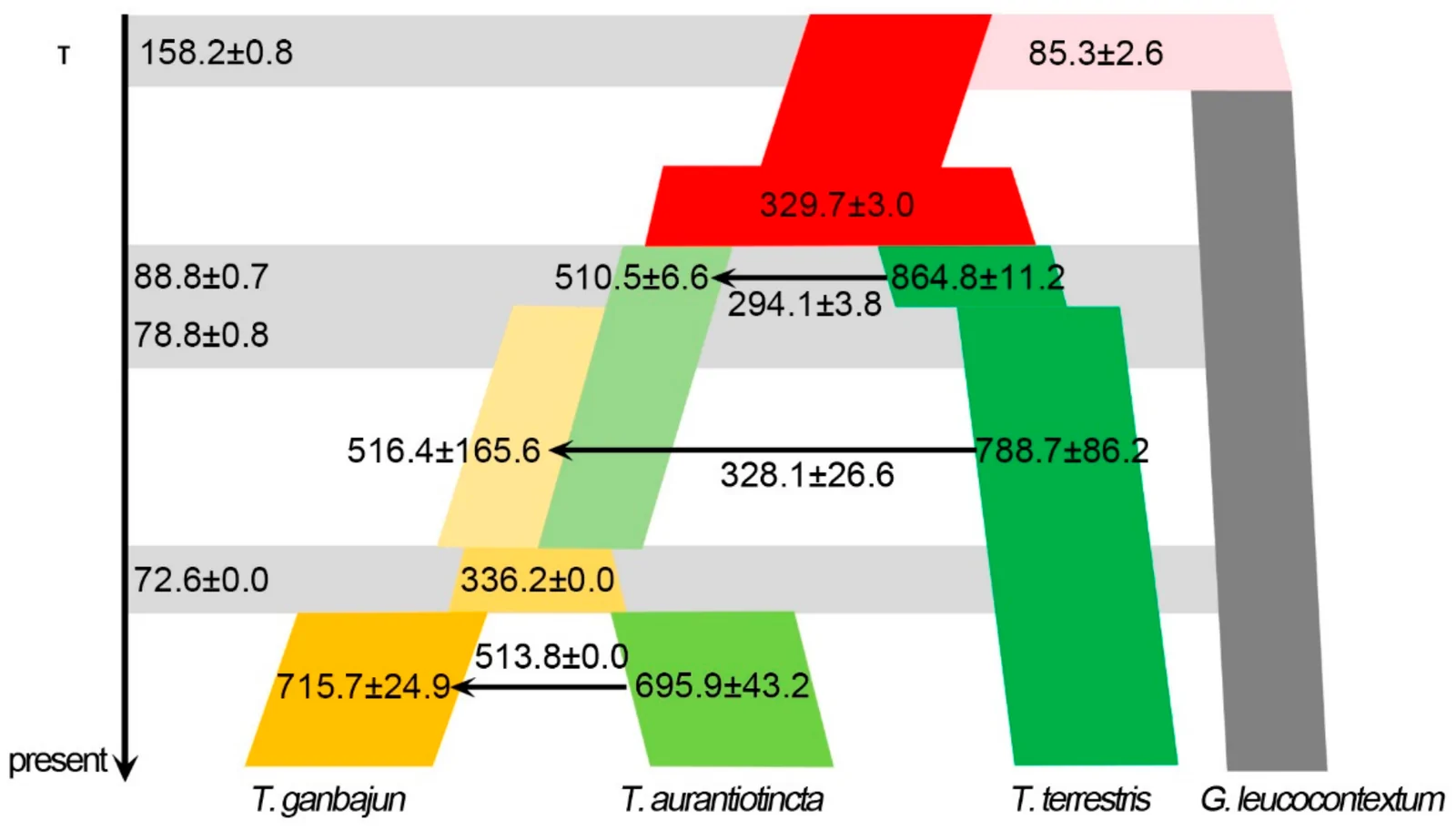
Gene flow and possible speciation processes in *Thelephora*. τ is the relative divergence time, measured by the expected number of mutations per site; θ is the population size parameter; and M is the expected number of migrants.

Gene flow analysis revealed complex patterns of historical genetic exchange among the three species (Figure 4). *T. terrestris* exhibited characteristics of unidirectional gene introgression as a genetic donor to the other two species. The migration rate from *T. terrestris* to *T. ganbajun* was 328.1 ± 26.6, while the reverse migration rate was near zero, with a corresponding divergence time of 78.8 ± 0.8. The migration rate from *T. terrestris* to *T. aurantiotincta* was 294.1 ± 3.8, with reverse migration of nearly zero and divergence time of 88.8 ± 0.7. The similar τ values of these two unidirectional introgression events suggested that *T. terrestris* contributed genetic variation to the ancestors of both *T. ganbajun* and *T. aurantiotincta* during similar historical periods. The migration rate from *T. aurantiotincta* to *T. ganbajun* was 513.8 ± 0.0, while the reverse migration was nearly zero, indicating unidirectional gene introgression from *T. aurantiotincta* to *T. ganbajun*. Effective population size analysis showed that the effective population size of the common ancestor of the three species was 329.7 ± 3.0, which contracted to 85.3 ± 2.6 for the common ancestor of *T. ganbajun* and *T. aurantiotincta*. Current effective population sizes of each species varied across different pairwise analyses: in the analysis of *T. ganbajun* and *T. terrestris*, effective population sizes were 516.4 ± 165.6 for *T. ganbajun* and 788.7 ± 86.2 for *T. terrestris*; in the analysis of *T. terrestris* and *T. aurantiotincta*, effective population sizes were 864.8 ± 11.2 for *T. terrestris* and 510.5 ± 6.6 for *T. aurantiotincta*; and in the analysis of *T. aurantiotincta* and *T. ganbajun*, effective population sizes were 715.7 ± 24.9 for *T. ganbajun* and 695.9 ± 43.2 for *T. aurantiotincta*, respectively.

## 4. Discussion

### 4.1. Phylogenetic Relationships and Ecological Niche Differentiation

The three *Thelephora* species form distinct lineages and are sister to the genus Sarcodon. Within the genus, the widespread *T. terrestris* diverged first (21.16 Mya, 95% HPD: 11.41–30.97 Mya), while *T. ganbajun* and *T. aurantiotincta* diverged from each other more recently (14.97 Mya, 95% HPD: 6.02–23.66 Mya). The two species restricted to southwestern China are therefore sister taxa, with the widespread species diverging earlier. This topology separates the ecological differences among the three species into two levels: divergence between the sister species, and divergence between them and the widespread species.

The two levels differ in the environmental axes along which the species separate. *T. ganbajun* and *T. aurantiotincta* occupy almost identical annual mean temperatures (medians 15.17 °C and 15.79 °C, with largely overlapping ranges) and are separated not by temperature per se but by temperature seasonality (median Bio4 = 452 and 760) and elevation (medians 1908 m and 461 m): the former is confined to climatically stable mid-elevations, the latter to low elevations with pronounced temperature fluctuation. Their suitable habitats overlap by 197,773 km^2^ in Yunnan, corresponding to 55% of the total suitable area of *T. ganbajun*. This extensive niche overlap, separated only along elevation and seasonality, is consistent with their peripatric distribution. *T. terrestris*, by contrast, differs from both along all major temperature and precipitation axes (median annual mean temperature 4.13 °C, minimum temperature of the coldest month down to −28.19 °C, median annual precipitation 564 mm), spans subtropical to cold-temperate zones, and contacts the two southwestern species only marginally. Divergence between the sister species thus occurs on a considerably narrower environmental scale than the earlier divergence within the genus.

Differences in genome size derive from repetitive sequences rather than gene number. *T. terrestris* has the largest genome (40.73 Mb) and the highest repeat content (26.79%), with an LTR content (13.41%) three times that of *T. ganbajun* (4.43%) and nine times that of *T. aurantiotincta* (1.46%). In the sequenced *T. terrestris* isolate, however, LTR insertions are concentrated within the last 0.5 Mya, more than an order of magnitude later than the divergence of this species at approximately 21 Mya. This expansion is therefore unrelated to the divergence of *T. terrestris* itself and occurred long after the species was established. Large-scale transposable element expansions can occur at the population level [68], and given that *T. terrestris* is a widely distributed species complex, the extent to which this burst is representative of the species cannot be determined from a single isolate.

The spatial relationship between transposable elements and genes is consistent across the three genomes: lineage-restricted genes are strongly enriched in transposable element-dense regions, particularly near LTR retrotransposons, whereas core genes, positively selected genes, and rapidly evolving genes tend to occur in transposable element-sparse regions. This pattern is present in all three species despite an almost tenfold difference in LTR content, indicating that different gene categories occupy different genomic environments with respect to transposable element density, and this difference does not depend on the absolute abundance of transposable elements. Within this pattern, the expanded gene families of *T. ganbajun* stand out: their median distance to the nearest transposable element is two orders of magnitude greater than for any other gene category in this species, which may indicate that these expansions are physically clustered in repeat-poor regions, more consistent with tandem duplication than with transposable-element-mediated dispersal; we note, however, that this estimate cannot exclude an influence of contig fragmentation, since distances were not measured across contig boundaries.

### 4.2. Functional Gene Differentiation

The expanded gene families of the three species fall into different functional categories. In *T. ganbajun*, expansions are concentrated in cell surface and ion homeostasis functions. Copper-related enrichment includes P-type divalent copper transporter activity (GO:0043682), copper ion homeostasis (GO:0055070), and monoatomic cation transport (GO:0006812) [69,70,71]. Its expanded CAZyme families, GH23, GT2, and CBM50, all act on microbial cell wall polysaccharides: GH23 degrades bacterial peptidoglycan [72], GT2 in fungi mainly encodes chitin synthases [73], and CBM50 (the LysM domain) binds chitin and chitooligosaccharides [74]. Yunnan is reported to be among the Chinese provinces with elevated soil copper content (National Earth System Science Data Center, https://www.geodata.cn accessed on 1 December 2025), and this provincial-level pattern overlaps the suitable range of *T. ganbajun*; however, we did not extract soil geochemical data at the resolution of our occurrence records, and this correspondence remains a coarse, untested observation rather than evidence that soil copper levels are the selective agent behind the expansion of copper-related gene families. In *T. terrestris*, expansions involve oxidative metabolism and stress response. Cytochrome P450-related enrichment includes heme binding (GO:0020037), iron ion binding (GO:0005506), and monooxygenase activity (GO:0004497); this system participates in oxidative stress response, membrane lipid regulation, and secondary metabolism in fungi. Protein phosphorylation signalling (GO:0006468, GO:0004674) is involved in environmental sensing and metabolic regulation. Its rapidly evolving genes are enriched in autophagosome assembly (GO:0000045) and cellular response to starvation (GO:0009267), and its positively selected genes include Hsp20 and DnaJ. These categories are directionally consistent with the temperature and precipitation range spanned by this species (Section 4.1). These categories are directionally consistent with the temperature and precipitation range spanned by this species; we report this correspondence descriptively and do not infer that these environmental conditions caused the expansion of the corresponding gene families. In *T. aurantiotincta*, expansions are concentrated in fatty acid metabolism, including medium-chain fatty acid-CoA ligase activity (GO:0031956), FAD binding (GO:0071949), methylation (GO:0032259), and phosphatidylcholine biosynthesis (GO:0006656). Of the two sister species, *T. aurantiotincta* occupies a range with markedly higher temperature seasonality (median Bio4 = 760 versus 452); we report this correspondence descriptively and do not infer from it that temperature seasonality is the cause of the fatty acid metabolism gene expansion in this species.

The CAZyme repertoires expanded in *T. ganbajun* and *T. terrestris* act on different substrate classes—microbial versus plant cell wall polysaccharides, respectively—and this contrast is consistent with, but does not on its own establish, a difference in ecological role between the two species. The families expanded in *T. ganbajun* act on microbial cell wall polysaccharides (chitin, peptidoglycan), whereas those expanded in *T. terrestris* all act on plant cell wall polysaccharides: CBM13 binds xylan and other carbohydrates, CE5 removes acetyl modifications from hemicellulose, and AA9 is a lytic polysaccharide monooxygenase acting on cellulose and some hemicellulose components [75,76,77]. We present these as hypotheses rather than conclusions. Testing them will require genome resequencing of multiple individuals, in situ environmental measurements at collection sites, and functional experiments on the relevant gene families.

### 4.3. Gene Flow

Likelihood ratio tests rejected the null hypothesis of strict isolation in all three species pairs (2ΔlnL = 30.72–66.51, *p* < 0.05), indicating historical genetic exchange among the three species.

Introgression was unidirectional in every comparison, and the directions were consistent: *T. terrestris* into both *T. ganbajun* and *T. aurantiotincta*, and *T. aurantiotincta* into *T. ganbajun*, with reverse migration negligible in all three comparisons. This direction corresponds exactly to the order of niche breadth among the three species. *T. terrestris*, with the broadest niche, was the donor in both comparisons in which it participated and never the recipient; *T. ganbajun*, with the narrowest niche, was the recipient in both of its comparisons and never the donor; and *T. aurantiotincta*, intermediate in niche breadth, received from *T. terrestris* and donated to *T. ganbajun*.

The niche relationship between the two sister species is consistent with this direction. The environmental tolerance of *T. aurantiotincta* encompasses that of *T. ganbajun* in temperature and precipitation: their annual mean temperature ranges almost coincide (11.01–19.11 °C versus 11.19–19.11 °C), but *T. aurantiotincta* tolerates colder extremes (minimum temperature of the coldest month −5.52 °C versus −2.66 °C); its annual precipitation range (787–1652 mm) extends beyond that of *T. ganbajun* (812–1458 mm) at both ends, and its elevational range extends far below the lower limit of *T. ganbajun* (32 m versus 1360 m), falling short only at the upper limit (2058 m versus 2514 m). The two species overlap by 197,773 km^2^ in Yunnan, corresponding to 55% of the suitable range of *T. ganbajun*. This largely nested relationship implies that individuals of *T. aurantiotincta* can persist under most conditions occupied by *T. ganbajun*, whereas the converse does not hold.

The τ estimates in the two comparisons involving *T. terrestris* were similar (78.8 and 88.8), consistent with the phylogeny: *T. terrestris* diverged from the common ancestor of the other two species, so its divergence times from each of them should be identical.

The three species have retained distinct niches and genome features despite historical genetic exchange. In each of the three pairwise comparisons, the direction of inferred introgression happened to align with the relative difference in environmental tolerance between the two species. This pattern is based on only three pairwise comparisons among three species, and is conditional on the assumptions of the isolation-with-migration model.

The three *Thelephora* species examined here occupy distinct environments, form separate genomic lineages, and differ in genome architecture and gene family content, with a history of unidirectional gene flow among them. These results document a pattern of ecological and genomic divergence together with historical gene flow in this group; whether the observed divergence was driven by ecological selection remains a hypothesis for future testing.

### 4.4. Limitations

Only one genome per species was analysed here, so intraspecific genomic variation could not be assessed. The distribution models are based on climatic and topographic variables, and the modelled ranges are conditional on the variable set used here. The forest mask used in this study distinguishes only among coniferous, broadleaf, and mixed forest types at a coarse scale and does not resolve host-tree distributions at the species level. In addition, the occurrence records used here are based on above-ground fruiting bodies, which may not fully reflect the below-ground distribution of ECM mycelia and root tips. Differences among the three genomes may reflect species-level divergence, or may reflect features of the sequenced isolates or their local populations, rather than adaptive divergence. Gene family expansion and contraction are based on gene counts and are therefore sensitive to assembly completeness, which differs among the three assemblies analysed here; contraction estimates are the most affected, since gene absence in an incomplete assembly cannot be distinguished from gene loss, and the gene count and LTR content reported for *T. aurantiotincta* may both be underestimates for this reason. Lineage-restricted genes are defined relative to the eleven genomes analysed here and are not necessarily absent from other fungi. Divergence times depend on three fossil calibration points and should be interpreted as approximate. Gene flow estimates rely on the isolation-with-migration model, which assumes constant effective population size and no recombination within a locus; departures from these assumptions, particularly unmodelled population size change, can produce migration signals that resemble the pattern reported here. Functional inferences rest on homology-based annotation and enrichment analysis rather than functional validation. The genomic differences described here may reflect neutral evolutionary processes or historical contingencies unrelated to the species’ current ecological niches, rather than adaptation, and the present data cannot distinguish between these possibilities. Testing the hypotheses proposed here will require genome resequencing of multiple individuals, in situ environmental measurements at collection sites, and functional experiments on the relevant gene families.

## Figures and Tables

**Table 1:** Environmental characteristics of suitable habitats for three *Thelephora* species.

Code	Environmental Variables	Unit	*T. ganbajun*	*T. aurantiotincta*	*T. terrestris*
			Minimum/Median/Maximum		
Bio1	Annual *mean* temperature	°C	11.19/15.17/19.11	11.01/15.79/19.11	−3.32/4.13/15.05
Bio2	Mean diurnal range (mean of monthly (max-min temp))	°C	9.13/10.77/11.74	7.12/8.20/10.86	8.24/12.07/14.10
Bio3	Isothermality ((Bio2/Bio7) × 100)		24.55/47.29/50.53	24.55/28.02/45.71	23.51/28.50/41.48
Bio4	Temperature seasonality (standard deviation × 100)	°C × 100	386/452/549	470/760/947	616/975/1540
Bio5	Maximum temperature of warmest month	°C	21.62/24.64/28.16	23.47/30.13/32.48	13.29/25.73/29.99
Bio6	Minimum temperature of coldest month	°C	−2.66/1.67/5.64	−5.52/0.91/5.81	−28.19/−18.57/0.24
Bio7	Temperature annual range (Bio5-Bio6)	°C	21.22/23.13/25.00	22.90/28.50/34.15	26.52/37.91/53.75
Bio8	Mean temperature of wettest quarter	°C	17.12/20.04/22.87	18.25/22.31/26.42	6.99/18.77/24.16
Bio9	Mean temperature of driest quarter	°C	4.29/9.14/14.35	0.74/6.72/13.19	−19.06/−9.31/5.43
Bio10	Mean temperature of warmest quarter	°C	17.12/20.04/22.91	18.80/25.26/27.52	7.03/19.09/24.91
Bio11	Mean temperature of coldest quarter	°C	4.06/9.02/13.38	0.50/5.93/11.61	−19.74/−9.81/5.33
Bio12	Annual precipitation	mm	812/1008/1458	787/1201/1652	332/564/1079
Bio13	Precipitation of wettest period	mm	154/199/298	168/216/296	84/140/213
Bio14	Precipitation of driest period	mm	7/12/16	8/20/43	2/4/16
Bio15	Precipitation seasonality (coefficient of variation)	—	70.15/84.22/90.16	51.75/66.67/93.52	69.28/98.30/118.30
Bio16	Precipitation of wettest quarter	mm	421/550/803	418/564/788	211/346/540
Bio17	Precipitation of driest quarter	mm	28/45/62	29/71/167	6/15/57
Bio18	Precipitation of warmest quarter	mm	414/549/797	410/514/704	209/343/526
Bio19	Precipitation of coldest quarter	mm	28/47/67	29/73/197	6/15/60
ELE	Elevation	m	1360/1908/2514	32/461/2058	122/1039/4451

Note: Bio4 (Temperature Seasonality) is calculated as the standard deviation of monthly mean temperatures × 100. Bio15 (Precipitation Seasonality) is the coefficient of variation of precipitation, dimensionless.

## Data Availability

The genome assembly of *T. ganbajun* has been deposited in the figshare database (DOI: https://doi.org/10.6084/m9.figshare.32593176). The pipeline scripts used to calculate the distances between transposable elements (TEs) and coding genes have been deposited on figshare for public access (DOI: https://doi.org/10.6084/m9.figshare.33328083). The geographic distribution records of the three *Thelephora* species, the gene annotation files of the three genomes, the analysis scripts, the ITS sequences and voucher specimen numbers of the collections examined, and the raw sequencing reads have all been deposited in the figshare database (DOI: https://doi.org/10.6084/m9.figshare.33363655).
